# Ligand-Induced Tyrosine Phosphorylation of Cysteinyl Leukotriene Receptor 1 Triggers Internalization and Signaling in Intestinal Epithelial Cells

**DOI:** 10.1371/journal.pone.0014439

**Published:** 2010-12-28

**Authors:** Ladan Parhamifar, Wondossen Sime, Yuliana Yudina, Frederik Vilhardt, Matthias Mörgelin, Anita Sjölander

**Affiliations:** 1 Cell and Experimental Pathology, Department of Laboratory Medicine, Clinical Research Center, Lund University, Skåne University Hospital, Malmö, Sweden; 2 Institute of Cellular and Molecular Medicine, Panum Institute, Copenhagen University, Copenhagen, Denmark; 3 Infectious Medicine, Department of Clinical Science, Lund University, Lund, Sweden; Universidade Federal do Rio de Janeiro, Brazil

## Abstract

**Background:**

Leukotriene D_4_ (LTD_4_) belongs to the bioactive lipid group known as eicosanoids and has implications in pathological processes such as inflammation and cancer. Leukotriene D_4_ exerts its effects mainly through two different G-protein-coupled receptors, CysLT_1_ and CysLT_2_. The high affinity LTD_4_ receptor CysLT_1_R exhibits tumor-promoting properties by triggering cell proliferation, survival, and migration in intestinal epithelial cells. In addition, increased expression and nuclear localization of CysLT_1_R correlates with a poorer prognosis for patients with colon cancer.

**Methodology/Principal Findings:**

Using a proximity ligation assay and immunoprecipitation, this study showed that endogenous CysLT_1_R formed heterodimers with its counter-receptor CysLT_2_R under basal conditions and that LTD_4_ triggers reduced dimerization of CysLTRs in intestinal epithelial cells. This effect was dependent upon a parallel LTD_4_-induced increase in CysLT_1_R tyrosine phosphorylation. Leukotriene D_4_ also led to elevated internalization of CysLT_1_Rs from the plasma membrane and a simultaneous increase at the nucleus. Using sucrose, a clathrin endocytic inhibitor, dominant-negative constructs, and siRNA against arrestin-3, we suggest that a clathrin-, arrestin-3, and Rab-5-dependent process mediated the internalization of CysLT_1_R. Altering the CysLT_1_R internalization process at either the clathrin or the arrestin-3 stage led to disruption of LTD_4_-induced Erk1/2 activation and up-regulation of COX-2 mRNA levels.

**Conclusions/Significance:**

Our data suggests that upon ligand activation, CysLT_1_R is tyrosine-phosphorylated and released from heterodimers with CysLT_2_R and, subsequently, internalizes from the plasma membrane to the nuclear membrane in a clathrin-, arrestin-3-, and Rab-5-dependent manner, thus, enabling Erk1/2 signaling and downstream transcription of the *COX-2* gene.

## Introduction

Patients with prolonged inflammatory conditions such as inflammatory bowel disease (IBD) exhibit increased levels of inflammatory mediators, such as cysteinyl leukotrienes (CysLT; LTC_4_, LTD_4_, and LTE_4_) [1]. The fact that IBD patients have a 30–50% increased risk of developing colorectal cancer [2] implies a possible role of cysteinyl leukotrienes in the coupling between chronic inflammation and the development of colorectal cancer.

Leukotrienes exert their effects through G-protein-coupled receptors (GPCRs). The CysLT_1_R [3] is a high affinity GPCR for the pro-inflammatory mediator LTD_4_ that is implicated in many inflammatory conditions [4], [5]. We have shown that LTD_4_ up-regulates several proteins related to carcinogenesis, such as COX-2, β-catenin, and Bcl-2, via the CysLT_1_R in intestinal epithelial cells [6], [7]. We have also shown that LTD_4_ mediates survival [8], [9], proliferation [10], and migration [11] in epithelial cells through the CysLT_1_R. Up-regulation of the receptors at the plasma membrane and the nuclear membrane was shown in a colon cancer tissue microarray [12]. This up-regulation of the CysLT_1_R in colon cancer correlates with a poorer prognosis [12], [13], [14]. In contrast to this, increased levels of the CysLT_2_R, which is also located in the plasma and nuclear membrane, correlates with a better prognosis for patients with colon cancer [14], [15]. Furthermore, LTC_4_-induced activation of CysLT_2_R has been shown to promote differentiation of colon cancer cells [15], which suggests a potentially opposite role for the CysLT_2_R compared to the CysLT_1_R in the development or progression of colon cancer.

A key regulatory mechanism of GPCR signaling is internalization and trafficking. There are a limited number of publications studying the trafficking of the CysLT_1_R [16], [17], [18]. Naik et al. demonstrated that in HEK-293 cells over expressing the CysLT_1_R, the internalization of the receptor is Protein Kinase C (PKC)-dependent [16]. Furthermore, our group has demonstrated that the nuclear localization sequence (NLS) domain, which contains the PKC sites, is required for internalization and Erk1/2 signaling via the CysLT_1_R [12]. Capra et al. showed that, unlike the homologous desensitization induced by LTD_4_, the heterologous desensitization of the CysLT_1_R via the P2YR is PKC-dependent [16], [17], suggesting that CysLT_1_R regulation can be cell specific. Previous results from our laboratory suggest that, upon stimulation with LTD_4_, the CysLT_1_R translocates from the plasma membrane to the outer nuclear membrane of Int 407 cells [12]. The internalization and trafficking of GPCRs are often implicated in GPCR-related pathologies, such as in the case of retinitis pigmentosa, which is reported to be a result of improper intracellular trafficking and localization of rhodopsin receptors [19], [20].

An important aspect of GPCR regulation is the ability to dimerize. GPCRs can induce signals as hetero-, homo-dimers or oligomers [21]. Moreover, GPCR dimerization has been shown to be needed for their proper expression, stronger ligand binding, phosphorylation, and internalization [21]. Dimerized GPCRs may have signaling properties distinct from those of monomeric receptors [22], [23]. Receptor-mediated endocytosis is a mechanism by which the cell regulates the magnitude and duration of external stimuli [24], [25]. There have been extensive investigations into endocytosis via clathrin-coated pits, resulting in it being the best-characterized mechanism for GPCR internalization [26], [27]. Clathrin-coated pits are membrane invaginations coated with clathrin. Upon ligand binding, G-protein-coupled receptor kinases (GRKs) or protein kinases, such as PKC, phosphorylate GPCRs. This phosphorylation leads to the recruitment of arrestin, which, in turn, targets the GPCR to the clathrin-coated pits. However, certain GPCRs, such as the leukotriene B_4_ receptor 1 (BLT_1_R), when transfected into Cos-7 and HEK-293 cells, may internalize independently of arrestins [28]. Different Rab proteins are involved in vesicle trafficking and regulate their directionality. Rab-5, -11 and -21, in particular, are involved in the trafficking of early endosomes [29], [30], [31]. Once internalized, the receptor is either recycled through early endosomes, sent for degradation to the lysosomes [32], or transported to the nucleus [33], [34], [35]. A less studied internalization pathway is the one through caveolae. Caveolae are membrane invaginations, rich in caveolin proteins and cholesterol. Certain GPCRs, such as the M1 receptor and the glucagon peptide 1 receptor, are internalized and have been shown to be internalized via this pathway [36], [37]. However, the mechanism of how these GPCRs are targeted into the caveolae is still unknown. Other GPCRs, like the β-adregenic receptors β_1_AR, and β_2_AR, are enriched in the caveolae, but they are not internalized through this pathway [38], [39].

The aim of this study was to investigate the underlying regulatory mechanisms leading to the internalization of CysLT_1_R. We demonstrate that LTD_4_ induces tyrosine phosphorylation and internalizes the CysLT_1_R. Furthermore, we suggest that the LTD_4_-induced CysLT_1_R translocation to the nucleius, or disruption of this internalization at various stages, could affect its overall signaling process.

## Materials and Methods

### Chemicals

Antibodies against heavy-chain clathrin were from BD Transduction Laboratories (Erembodegem, Belgium). The LTD_4_ was from Cayman Chemical Company (Ann Arbor, MI), and N-terminal CysLT_1_R was from Innovagen (Lund, Sweden). The ZM198615 was a gift from AstraZeneca (R&D, Lund, Sweden), and ECL Western blot detection reagents and Hyperfilm were from Amersham International (Buckinghamshire, UK). The source for Protein A sepharose was GE Healthcare (Uppsala, Sweden). The phospho-Erk1/2 antibody was from New England BioLabs Inc, (Beverly, MA). Antibodies against arrestin-3 were purchased from Cell Signaling (Boston, MA) and Santa Cruz (Santa Cruz, CA). The arrestin-3 and scrambled siRNA were from Santa Cruz (Santa Cruz, CA). Peroxidase-linked goat anti-rabbit antibodies and fluorescence mounting medium were from Dako A/S (Copenhagen, Denmark). Lipofectamine 2000 and all cell culture media were from Invitrogen (Carlsbad, CA) and Alexa 488 and Alexa 546 were from Molecular Probes Inc. (Leiden, Netherlands). The RNeasy MinElute Spin Column was from Qiagen (Hilden, Germany). Genistein was from Calbiochem (San Diego, CA). The Flag M2 antibodies, light-chain clathrin antibodies, and all other chemicals were of analytical grade and were purchased from Sigma Chemical Company (St. Louis, MO).

### Cell Culture

Non-transformed human intestinal epithelial cells, Int 407 cells exhibiting typical epithelial growth and morphology [40], and the human colorectal adenocarcinoma cell line Caco-2 (ATCC HTB-37) were cultured as described previously [41]. Cells were cultured to approximately 80% confluency and regularly tested to ensure the absence of mycoplasma.

### The *in situ* proximity ligation assay

The *in situ* proximity ligation assay (PLA), Duolink™, was from Olink Bioscience (Uppsala, Sweden) and performed according to the manufacturer's instructions [42], with slight modifications. Briefly, Int 407 cells were grown in 4-well plates to 50% confluency, serum starved, and stimulated with LTD_4_ or LTC_4_ (40 nM) for indicated time points and fixed for 15 minutes in 4% ice-cold PFA/PBS. Blocking in a 3% BSA/PBS for 1 hour followed. Thereafter, the cells were stained with anti-rabbit CysLT_1_R, anti-goat CysLT_2_R antibodies, and anti-phospho-tyrosine antibodies (1∶250 in 3% BSA/PBS) overnight at 4°C. This was followed by washing five times in PBS-T and incubation with PLA probes minus and plus (anti-goat DNA minus strand and anti-rabbit DNA-plus strand, diluted 1∶5) in 3% BSA/PBS for 2 hours at 37°C. Alternatively, as a negative control, the CysLT_2_R antibody, or the DNA-plus probe, was omitted. Furthermore, as a positive control, the Duolink control kit contains two primary antibodies targeting different epitopes of chicken TK1 protein. Thereafter, the cells were washed twice in PBS-T and hybridized at 37°C for 15 minutes, followed by ligation for 15 minutes at 37°C. The cells were washed 1× in PBS-T and treated with polymerase for amplification for 90 minutes at 37°C. The detection of PLA-amplicons (red dots) was carried out using the “563 detection kit” provided by Olink Bioscience. This kit includes the Hoechst 33342 dye for nuclear staining (blue) and the Alexa Fluor 488-phalloidin/actin for cytoplasmic staining (green). The cells were then mounted and examined using a Nikon TE300 microscope (60×1.4 plan apochromat oil immersion objective), integrated into fluorescent microscopy. The red dots were counted using the MATLAB/Blob Finder software from Olink Bioscience (Uppsala, Sweden) [42].

### Electron Microscopy

Cells stimulated with or without 40 nM LTD_4_ or LTC_4_ for 15 or 30 minutes were used for electron microscopy and prepared as described previously [12]. Briefly, 5×10^6^ cells were pelleted at 4°C immediately after being placed in a fixative (4% paraformaldehyde and 0.1% glutaraldehyde). The pellets were dehydrated in ethanol for 1 hour at room temperature and then embedded in Lowicryl [43]. Ultra thin sections were cut on a microtome and mounted on nickel grids. For immunostaining, the grids were floated on drops of immune reagents placed on a sheet of parafilm. Free aldehyde groups were then blocked with 50 mmol/L glycine, and the grids were subsequently incubated with 5% (v/v) donkey serum in PBS supplemented with 0.2% bovine serum albumin (BSA; pH 7.6) for 15 minutes. Overnight incubation with the primary antibody (dilution 1∶100) at 4°C followed this blocking procedure. The grids were subsequently washed by placing them, successively, on 10 drops of incubation buffer (5 minutes on each drop), after which the sections were incubated with the gold-conjugated secondary antibody by letting them float on drops containing the gold conjugate reagent (diluted 1∶20 in incubation buffer) for 60 minutes at room temperature. After further washing on 10 drops of incubation buffer, the sections were postfixed in 2% glutaraldehyde. Finally, the sections were washed with distilled water, poststained with uranyl acetate and lead citrate, and examined using a Jeol 1200 EX transmission electron microscope operated at 60 kV accelerating voltage, as previously described [12]. The antibody directed against the CysLT_1_ was labeled with 10-nm colloidal thiocyanate gold and CysLT_2_ with 5-nm colloidal thiocyanate gold [44] for the LTC_4_ experiment. But, 10-nm colloidal thiocyanate gold for CysLT_2_R and 5-nm colloidal thiocyanate gold for CysLT_1_R in the LTD_4_ experiment. The images were recorded with a Gatan Multiscan 791 CCD camera. Researchers examined sixty cellular profiles for evaluation.

### Immunofluorescent Staining

Cells were grown on cover slips to 50–60% confluency, pre-treated, or not, with ZM198,615 (40 µM, 15 minutes) and then treated with, or without, LTD_4_ (80 nM, 5 minutes or as indicated). Cells were washed once and kept in 1.5% serum-containing medium for 15 or 20 minutes. Thereafter, the cells were fixed for 15 minutes in 4% PFA/PBS, followed by blocking in a 3% BSA/PBS for 1 hour for anti-CysLT_1_R, 5% goat serum and 1% TritonX100/PBS for anti-Flag, or 3% milk/PBS for anti-clathrin. Cells were then incubated overnight with anti-CysLT_1_R (1∶250) in a 3% BSA/PBS or 1% goat serum in PBS-Tween (PBS-T) for Flag (1∶2500), and clathrin (1∶250) in 2% BSA/PBS. Cells were washed five times in PBS and incubated for 1 hour with secondary antibody goat anti-rabbit IgG Alexa 488 or 546 (3% BSA/PBS 1∶250) for CysLT_1_R and clathrin or 1% goat serum 1∶800 for Flag antibodies. Following five washes with PBS, the cover slips were mounted on glass slides with a fluorescence-mounting medium and examined using a Nikon TE300 microscope (60×1.4 plan-apochromat oil immersion objective), integrated in fluorescent microscopy.

### Transfection

Cells were grown on cover slips to 50–60% confluency. Transfection was performed with GFP-DN-Eps-15, GFP-Eps-15, Flag-CysLT_1_R, or GFP-Rab-5 constructs, using lipofectamine according to the manufacturer's protocol. Briefly, cells were transfected for 6 hours in serum-free medium and left to rest for 48 hours in complete medium before analysis. For siRNA, cells were grown to about 60% confluency in 6-well plates, transfected in serum-antibiotic-free medium, with 80 pmol siRNA against arrestin-3 or scrambled siRNA using lipofectamine 2000. After 6 hours of transfection, 1 mL serum-free medium was added to each well and cells were left overnight. The medium was then changed to normal growth medium and cells were left to rest for an additional 48 hours before being lysed or used for FACS analysis.

### Cell Lysates, Immunoprecipitation and Fractionation

Cells were left in serum-free medium for 2 hours, pre-treated with Filipin (5 µg/mL, 1 hour), sucrose (0.4 M, 1 hour), or cycloheximide (100 µg/mL, 1 hour) and then treated with, or without, LTD_4_ (80 nM) for indicated time points. The stimulations were terminated by the addition of ice-cold lysis buffer A (20 mM sodium Hepes pH 8.0, 2 mM MgCl_2_, 1 mM EDTA, 5 mM sodium orthovandate, 60 µg/mL phenylmethylsulfonyl fluoride (PMSF), and 4 µg/mL leupeptin) and the cells were placed on ice. The cells were then scraped from the flasks. The supernatant was collected from the cell lysate preparation after a centrifugation at 200×g for 10 minutes at 4°C and after a centrifugation at 10,000×g for 15 minutes at 4°C. The samples were compensated to equal protein content and pre-cleared with 1 µg of rabbit IgG and 15 µl of protein A-sepharose for overnight at 4°C. The samples were immunoprecipitated with 5 µg of CysLT_2_R antibody for 2 hours at 4°C. Thereafter, 20 µg of protein A-sepharose beads were added, and the samples were rotated for an additional 1 hour at 4°C. The precipitates were washed multiple times with lysis buffer A. For fractionation, the cells were subjected to N_2_-decompression at 1,000 psi for 10 minutes, using a cell disruption bomb (Parr Instrument Company, Moline). The intact nuclei were collected by centrifugation at 200×g and washed twice in buffer A. The supernatant was centrifuged at 10,000×g for 10 minutes, and the resulting supernatant was fractioned into cytosol and plasma membrane fractions by centrifugation at 200,000 g for 1 hour.

### Gel Electrophoresis and Immunoblotting

Cell lysates were solubilized by boiling in sample buffer (62 mM Tris pH 6.8, 1.0% SDS, 10% glycerol, 15 mg/mL dithiothreitol, and 0.05% bromphenol blue), loaded, and subjected to electrophoresis on 10% homogeneous polyacrylamide gels. The separated proteins were electrophoretically transferred to PVDF membranes. The CysLT_1_R membranes were incubated overnight at 4°C with anti-CysLT_1_R and CysLT_2_R (diluted 1∶250 in 3% BSA/PBS) and for 1 hour at room temperature for anti-actin (1∶2000 in 2% BSA/PBS). After washing three times, the membranes were incubated for 1 hour at room temperature with HRP-conjugated secondary antibody (1∶5000 in 1% BSA/PBS for CysLT_1_R and CysLT_2_R or 1∶3000 in 1% BSA/PBS for actin), and then the membranes were washed three to six times. Thereafter, the membranes were incubated with ECL Western blot detection reagents and exposed to Hyperfilm-ECL to visualize immunoreactive proteins.

### DNA Isolation and Sequencing

Cells were grown to 80% confluency, scraped, and resuspended in lysis buffer (50 mM Tris-HCl, pH 8.0, 15 mM EDTA, 200 mM NaCl, and 0.5% SDS). The mixture was incubated overnight at 45°C with Proteinase K (Fermentas, Vilnius, Lithuania). Phenol was added and mixed for 10 minutes. The mixture was centrifuged at 600 g for 10 minutes at 10°C. The upper clear aqueous layer was carefully transferred to a new tube. An equal volume of phenol∶chloroform∶isoamyl alcohol (24∶24∶1) was added and mixed, by gentle inversion, for about 10 minutes and centrifuged at 500 g for 10 minutes at 10°C. The upper clear aqueous layer was transferred to a new tube. An equal volume of chloroform∶isoamyl alcohol (24∶1) was added, mixed for 10 minutes, and centrifuged at 500 g for 10 minutes at 10°C. The upper clear aqueous layer was transferred to a new tube. One-tenth of the volume of 3 M sodium acetate, pH 5.2, and double volumes of 100% isopropanol were added and allowed to stand for 1 hour at −20°C. The samples were centrifuged at 11,000 g for 10 minutes at 4°C thereafter. The supernatant was discarded and the pellet washed with 70% ethanol. The resultant pellet was dried and dissolved in TE-buffer (10 mM Tris, pH 8.0, 1 mM EDTA). The Department of Clinical Chemistry at Skåne University Hospital (SUS), Malmö, Sweden, performed the sequencing.

### RT-PCR

Cells were scraped on ice in PBS, homogenized 10 times with a 20 G needle, and then centrifuged for 2 minutes at 10,000 g. The pellet was resuspended in 1 ml TRIzol and immediately frozen at −80°C. The RNA was isolated using the phenol-chloroform extraction method. The RNA was dissolved in RNase-free H_2_O and purified on RNeasy MinElute Spin Columns. The cDNA synthesis was performed using Superscript™ II reverse transcriptase. Next, 2 µg of cDNA was mixed with 0.9 µM TaqMan primers and master mix and amplified at 60°C in a Mx3005P (Stratagene qPCR system). The following primer set was used: COX-2: Hs01573475_g1 and GAPDH: Hs00266705_g1. The samples were analyzed and normalized against a housekeeping gene (GAPDH) using the MX-Pro software (Stratagene).

### Flow Cytometry

Int 407 or Caco-2 cells were cultured as described previously; thereafter, they were serum-starved before they were either transfected with siRNA against arrestin-3 or scrambled siRNA and then stimulated with or without, 80 nM LTD_4_ for 5 or 30 minutes. The cells were detached by the addition of versen or trypsin-EDTA, respectively. The collected cells were first washed with cold PBS supplemented with 0.2 mM EDTA and then with PBS containing 0.5% bovine serum albumin. The cells (1×10^6^) were first fixed using the IC-Fixation buffer (cat # 00-8222; eBioscience, San Diego, Ca) before doing cell surface staining for CysLT_1_R or further permabilized for intracellular staining of arrestin-3 using the Permabilization Buffer (cat # 00-8333; eBioscience, San Diego, Ca). Following the recommendation given by the manufacturer and supplemented with additional washing steps, the cells were stained with the anti-CysLT_1_R primary antibody (5 µg/mL) or the anti human arrestin-3 antibody (5 µg/mL) followed by incubations with either goat anti-Rabbit IgG or goat anti-mouse IgG secondary antibody both conjugated respectively with ALEXA-488 (1∶100 in 0.5% BSA/PBS). An equivalent amount of non-specific rabbit or mouse IgG was used as controls. A single-color, immunofluorescence, flow cytometry analysis was performed on a FACSCalibur (Becton Dickinson) and data were analyzed using software (CellQuest; Becton Dickinson). Each measurement was based on the analysis of 10,000 cells.

### Statistical Analysis

Results are expressed as mean ± SEM. Differences between experimental groups were assessed by a Student's t test and one way ANOVA. P values of <0.05 were considered significant. *P<0.05 and ** P<0.01 and ***P<0.001.

## Results

### Hetrodimerization of the CysLTRs and Tyrosine Phosphorylation of the CysLT_1_R

**Figure 1:**
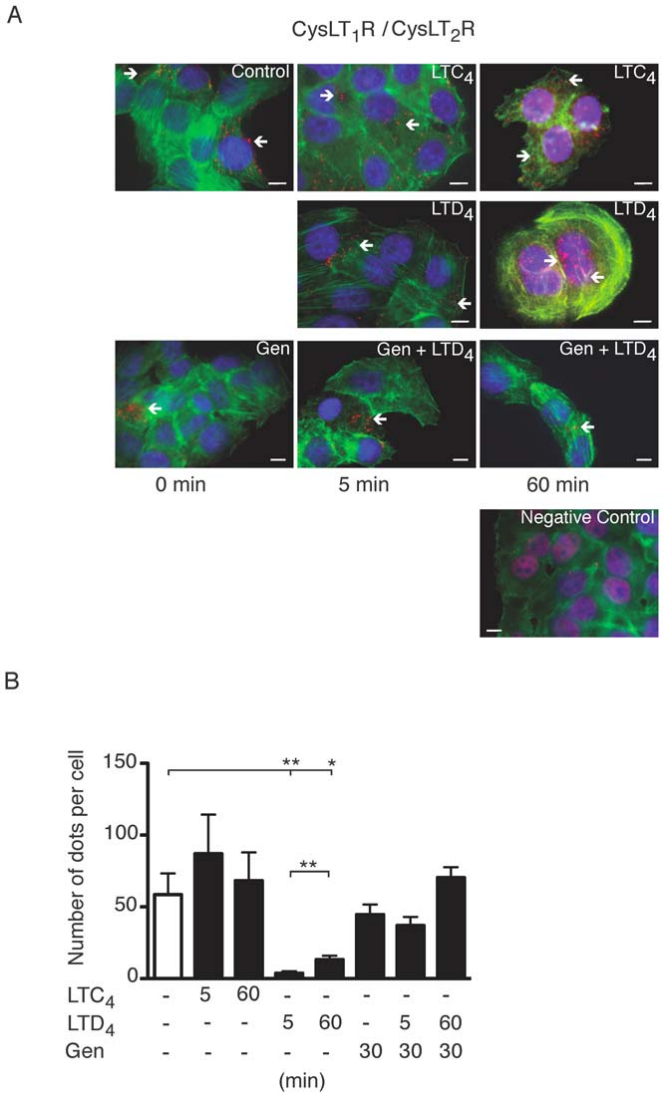
Receptor heterodimerization detected by a *in situ* proximity ligation assay (PLA). (**A**) Briefly, Int 407 cells were grown to 50% confluency, stimulated with or without LTD_4_ (40 nM), LTC_4_ (40 nM), or pre-incubation with genistein (50 µg/ml) for 30 minutes. The receptor interactions were studied employing PLA, treated according to the manufacturer's instructions using the CysLT_1_R antibody (1∶250) and the CysLT_2_R antibody (1∶250) and mounted on glass slides with a fluorescence-mounting medium. Alternatively, the CysLT_2_R antibody was omitted as a negative control. The mounted slides were examined using a Nikon TE300 microscope (60×1.4 plan apochromat oil immersion objective), integrated in fluorescent microscopy. The detection of PLA-amplicons (red dots) was carried out using the “563 detection kit”. This kit includes the Hoechst 33342 dye for nuclear staining (blue) and the Alexa Fluor 488-phalloidin/actin for cytoplasmic staining (green). The red dots indicate close proximity between cellular bound antibodies, and they were counted using the MATLAB/Blobfinder software. (**B**) The data are given as percent of control and represent means ± S.E.M. of at least three separate experiments. The statistical analysis was performed with a Student's *t* test. *P<0.05 and ** P<0.01. The scale bar represents 10 µm.

Because of the overlapping localization of the CysLT_1_R and CysLT_2_R at the plasma membrane and nuclear membrane, we investigated a potential heterodimerization of the receptors. Previous studies have demonstrated that the CysLT_1_R and CysLT_2_R might dimerize in mast cells [45]. Heterodimerization of the CysLT_1_R and CysLT_2_R was examined in Int 407 cells using the *in situ* proximity ligation assay (PLA) (Fig. 1). With this assay, protein-protein interactions *in situ* can be detected and visualized; when the secondary antibody is in close proximity, a fluorescent labeling of the DNA product is produced (red dots) [42]. Image analysis is based on counting the number of red dots/cell. The negative control without the CysLT_2_R antibody does not produce any red dots (Fig. 1A). Cells stained with both the CysLT_1_ and CysLT_2_ receptor antibodies showed that the receptors were heterodimerized under basal conditions (Fig. 1). The heterodimers (red dots) were concentrated to the plasma membrane and the nuclear region (Fig. 1A). Stimulation with LTC_4_ (40 nM) for 5 minutes caused a slight increase in the number of heterodimerized receptors, however this effect was not statistically significant (Fig. 1B). These results suggest that CysLT_1_R and CysLT_2_R are dimerized already, under basal conditions, and remain dimerized, even after LTC_4_ stimulation.

We found a statistically significant decrease in heterodimerization of the receptors 5 minutes after LTD_4_ stimulation (an average of less than 4 dots/cell compared to an average of 60 dots/cell in the control; Fig. 1B). However, after 60 minutes of LTD_4_ stimulation, a slight increase in heterodimerization (an average of 13 dots/cell) compared to the 5 minutes value was observed. Interestingly, the heterodimers (red dots) were mainly localized to the nuclear region. To further confirm the association between the receptors, we preformed immunoprecipitation with the CysLT_2_R antibody. With this approach we could confirm an association between the CysLT_2_R and the CysLT_1_R that was reduced (P<0.05) after LTD_4_ stimulation (Figure S1).

Both receptors contained several tyrosine phosphorylation sites, which might be important for activation and internalization. We therefore, investigated if tyrosine phosphorylation was involved in the decreased heterodimerization seen after LTD_4_ stimulation. Indeed, we found that the effect of LTD_4_ was abolished in cells pretreated with genistein, a broad phosphotyrosine inhibitor (Fig. 1B).

**Figure 2:**
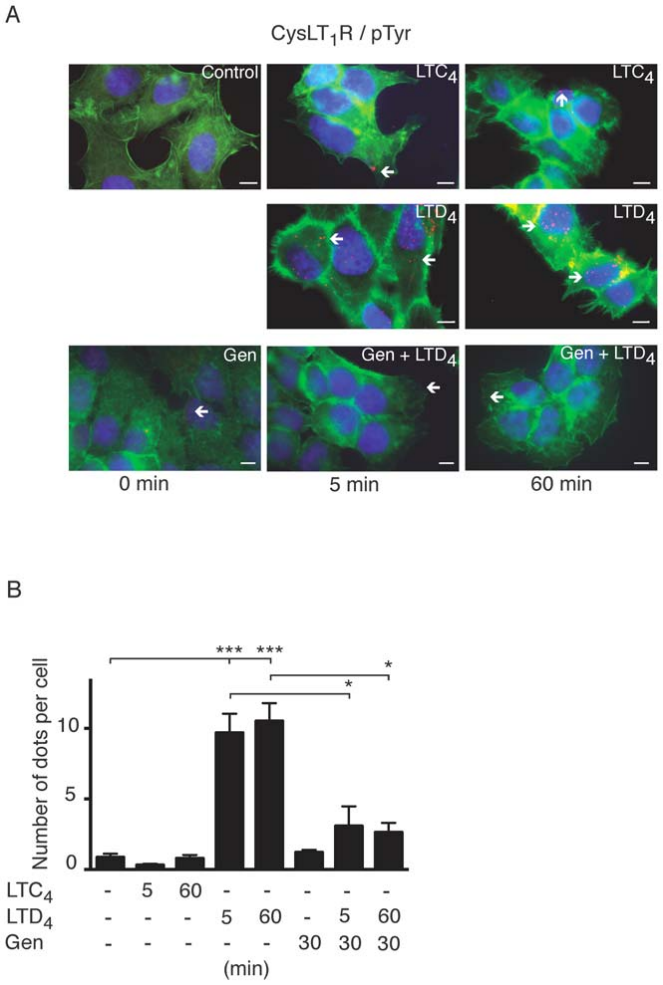
The CysLT_1_R tyrosine phosphorylation detected by in situ proximity ligation assay (PLA). Briefly, Int 407 cells were grown to 50% confluency, stimulated with or without LTD_4_ (40 nM), LTC_4_ (40 nM), or pre-incubation with genistein (50 µg/ml) for 30 minutes. The receptor tyrosine phosphorylation was studied employing PLA, treated according to the manufacturer's instructions using (**A**) the CysLT_1_R antibody (1∶250) and the phosphor-Tyr antibody (1∶250) and mounted on glass slides with a fluorescence-mounting medium. The mounted slides were examined using a Nikon TE300 microscope (60×1.4 plan apochromat oil immersion objective), integrated in fluorescent microscopy. The detection of PLA-amplicons (red dots) was carried out using the “563 detection kit”. This kit includes the Hoechst 33342 dye for nuclear staining (blue) and the Alexa Fluor 488-phalloidin/actin for cytoplasmic staining (green). The red dots indicate close proximity between cellular bound antibodies, and they were counted using the MATLAB/Blobfinder software. (**B**) The data are given as percent of control and represent means ± S.E.M. of at least three separate experiments. The statistical analysis was performed with a Student's *t* test. *P<0.05 and ** P<0.01. The scale bar represents 10 µm.

As demonstrated, LTD_4_ (40 nM) induced tyrosine phosphorylation of the CysLT_1_R after 5 and 60 minutes of stimulation (an average of 10 dots/cell as compared to 1 dot/cell when not stimulated), whilst LTC_4_ (40 nM) did not induce any detectable increase in tyrosine phosphorylation of the CysLT_1_R (Fig. 2B). Genistein significantly reduced the LTD_4_-induced tyrosine phosphorylation of CysLT_1_R (an average of 3 dots/cell; Fig. 2B). Neither LTC_4_ nor LTD_4_ induced any detectable tyrosine phosphorylation of the CysLT_2_R (data not shown). We also investigated threonine phosphorylation of the receptors with the PLA technique. For this experiment, we used an antibody for anti-phospho-threonine (Abnova Taiwan Corp), but we were not able to detect any threonine phosphorylation upon LTD_4_ or LTC_4_ stimulation of either of these receptors during the time points tested (data not shown).

### Internalization of the CysLT_1_R and the CysLT_2_R

**Figure 3:**
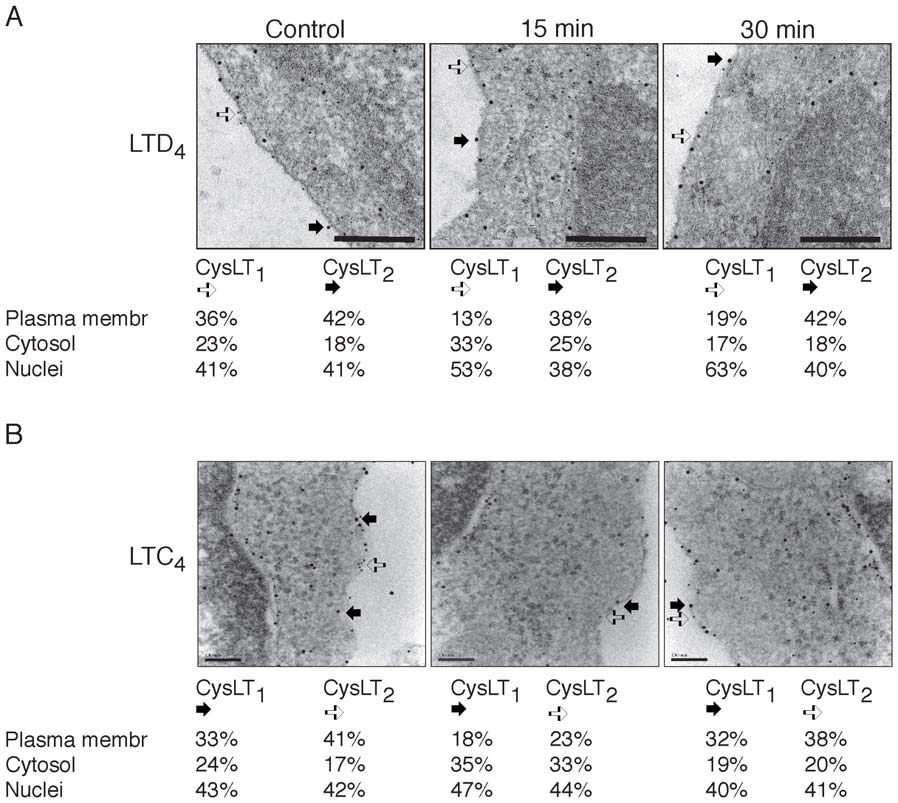
Electron microscopy images of CysLT_1_R and CysLT_2_R. Electron microscopy of Int 407 cells treated without or with (**A**) LTD_4_ (40 nM, 15 or 30 minutes) or (**B**) LTC_4_ (40 nM, 15 or 30 minutes). Samples of intact cells used for electron microscopy were prepared by pelleting 5×10^6^ cells immediately after adding a fixative (4% paraformaldehyde and 0.1% glutaraldehyde). Ultra thin sections were cut on a microtome and mounted on nickel grids, followed by overnight incubation with the primary antibody against CysLT_1_R and CysLT_2_R. (**A**) The antibody directed against the CysLT_1_R was labeled with 10-nm colloidal thiocyanate gold (black arrow) and CysLT_2_R with 5-nm colloidal thiocyanate gold (white arrow). The scale bar represents 0.2 µm. (**B**) The antibody directed against the CysLT_1_R was labeled with 5-nm colloidal thiocyanate gold (white arrow) and CysLT_2_R with 10-nm colloidal thiocyanate gold (black arrow). The scale bar represents 0.1 µm. The specimens were examined using a Jeol JEM 1230 electron microscope operated at 60 kV accelerating voltage, and images were recorded with a Gatan Multiscan 791 CCD camera.

We next examined the regulation of low affinity CysLT_2_R in conjunction with the CysLT_1_R. Int 407 cells were primarily stimulated with 40 nM LTD_4_ and receptor internalization and dimerization were visualized using electron microscopy (Fig. 3A). The CysLT_1_R was labeled with 10-nm colloidal thiocyanate gold particles and CysLT_2_R was labeled with 5-nm colloidal thiocyanate gold particles; as a result, both heterodimers and homodimers could be seen. Interestingly, upon LTD_4_ stimulation, it was mainly the CysLT_1_R that was internalized and localized to the nucleus, both after 15 minutes (53%) and 30 minutes (63%), compared to the CysLT_2_R (38% and 40%, respectively) (Fig. 3A). We also found a reduction of the CysLT_1_R at the plasma membrane in cells stimulated with LTD_4_ for 15 and 30 minutes (13 and 19%, respectively, as compared to 36% in the control). However, the majority of the CysLT_2_R did not internalize during this period. We then investigated the effect of LTC_4_ on receptor internalization (Fig. 3B). In these experiments, the CysLT_1_R was labeled with 5-nm colloidal thiocyanate gold particles and CysLT_2_R was labeled with 10-nm colloidal thiocyanate gold particles. Interestingly, both receptors internalized upon 15 minutes of 40 nM LTC_4_ stimulation, and this could be explained by the decrease from the plasma membrane and a small increase in the cystosol of both CysLT_1_R and CysLT_2_R (Fig. 3B). The receptor levels were restored to the plasma membrane again after 30 minutes of LTC_4_ stimulation (Fig. 3B). This was further confirmed by Western blot analysis of the plasma membrane fractions, showing a significant LTC_4_-induced decrease in the CysLT_1_R and CysLT_2_R expression in the plasma membrane after 5-15 minutes of stimulation, effects that are reversed after 30 minutes (Figure S2).

### Internalization and Recycling of the CysLT_1_R

**Figure 4:**
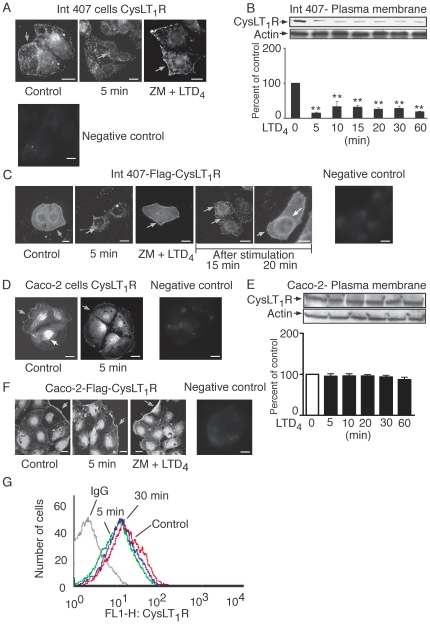
The localization and internalization of the CysLT_1_R in epithelial cells. Immunofluorescent staining of endogenous CysLT_1_R (**A, D**) and Flag-tagged CysLT_1_R (**C**, **F**) in Int 407 (**A**, **C**) and Caco-2 cells (**D**, **F**). Cells were grown on cover slips to 50–60% confluency pre-treated, or not, with ZM198,615 (40 µM, 15 minutes) and treated with or without 80 nM LTD_4_ for 5 minutes or as indicated. The cells were fixed and stained with CysLT_1_R antibody (1∶250) or Flag antibody (1∶2500) and mounted as described in Methods. The mounted slides were examined using a Nikon TE300 microscope (100×1.4 plan-apochromat oil immersion objective). The scale bar represents 10 µm. Int 407 cells (**B**) and Caco-2 cells (**E**) were grown to 80% confluency, serum-starved for 2 hours and treated with or without 80 nM LTD_4_ for indicated time points. Plasma membrane fractions were prepared as described in Methods and samples were subjected to SDS-polyacrylamide gel electrophoresis and Western blot analysis. The PDVF membranes were stained with the CysLT_1_R (1∶1000) and re-probed with actin (1∶2000) antibodies. The data are given as percent of control and represent means ± S.E.M. of at least three separate experiments. The statistical analysis was performed with a Student's *t* test. *P<0.05 and ** P<0.01. (**G**) A representative FACS analysis histogram overlay displaying the relative fluorescence intensity of CysLT_1_R surface expression is shown for Caco-2 cells.

Previous results from our lab have shown that the CysLT_1_R is localized to the plasma and nuclear membranes of intestinal epithelial and colon cancer cells [12]. In this study, we investigated how the CysLT_1_R is internalized and increased at the nuclear membrane. In agreement with our previous results, we demonstrated that the endogenous CysLT_1_R is localized to both the plasma membrane and nuclear region of unstimulated Int 407 cells using fluorescent microscopy (Fig. 4A). We also showed that, upon 5 minutes of stimulation with 80 nM LTD_4_, the CysLT_1_ receptor is rapidly internalized (Fig. 4A). The internalization is seen as intracellular punctuated dots (Fig. 4A), which can be blocked by pre-treatment with the CysLT_1_R antagonist ZM198,615 (40 µM, 15 minutes; Fig. 4A) or PKC inhibitor GF109203X (2 µM, 15 minutes; data not shown). The internalization was also confirmed by Western blot (Fig. 4B). We next transfected Int 407 cells with a Flag-tagged CysLT_1_R construct, stained with a Flag antibody. The distribution of Flag-tagged CysLT_1_R is more uniformly distributed than the endogenous receptor, most likely due to the Flag construct. However, the Flag-tagged CysLT_1_R was also localized to both the plasma membrane and nuclear region, similar to endogenous CysLT_1_R staining, as it was internalized after 5 minutes of LTD_4_ stimulation and could be significantly blocked by the specific CysLT_1_R antagonist, ZM198,615 (40 µM, 15 minutes; Fig. 4C). In summary, the endogenous and the over expressed Flag-tagged CysLT_1_R was localized to both the plasma membrane and the nuclear region of the cell, and was internalized after 5 minutes of LTD_4_ stimulation. Receptor recycling was a key mechanism regulating many different receptors [25]; therefore, the recycling of the CysLT_1_R was investigated. After 5 minutes of stimulation with LTD_4_, the ligand was removed by changing the medium to a LTD_4_-free growth medium for an additional 15–20 minutes before they were fixed and stained. The Flag-tagged CysLT_1_R recycled back to the plasma membrane 15–20 minutes after stimulation (Fig. 4C). We also investigated the endogenous receptor localization in Caco-2 cells, which showed a similar pattern, but no internalization could be detected after 5 minutes of stimulation with 80 nM LTD_4_ (Fig. 4D). We, therefore, performed Western blot analyses of the plasma membrane fractions of the Caco-2 cells. No significant decrease of the endogenous receptor after 5–60 minutes of LTD_4_ stimulation could be detected in Caco-2 cells (Fig. 4E). However, in cells transfected with the Flag-tagged CysLT_1_R, the receptor internalization could be detected after 20 minutes of LTD_4_ stimulation (Fig. 4F). This internalization was sensitive to pre-treatment with the receptor antagonist ZM198,615 (Fig. 4F). We investigated if the undetectable internalization of endogenous CysLT_1_R in Caco-2 cells was due to a mutation in the endogenous receptor of colon cancer cells; the CysLT_1_R from three colon-cancer cell lines (Caco-2, SW-480, and HCT-116) were sequenced. However, no mutation in the CysLT_1_R sequence was detected (data not shown).

We next performed FACS analysis of the endogenous CysLT_1_R in the colon cancer cell line, Caco-2, to confirm the CysLT_1_R transfection data with the endogenous receptor, using a more sensitive method. Figure 4G shows the overlay for FACS histograms of Caco-2 cells, where the CysLT_1_R internalization could be detected after 5 minutes of LTD_4_ stimulation. Other than the shift in histogram peaks, as shown in Figure 4G, we also evaluated the change in both median and mean fluorescent intensity of CysLT_1_R expression (data not shown). Based on the findings obtained from all the different approaches, our results confirmed that the CysLT_1_R is internalized in both colon cancer cells and non-transformed intestinal epithelial cells.

### Co-localization of Clathrin with the CysLT_1_R

**Figure 5:**
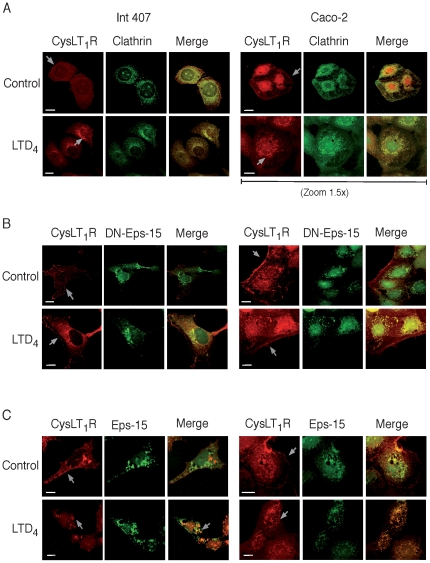
Expression and co-localization of clathrin or Eps-15 with the CysLT_1_R in Int 407 and Caco-2 cells. (**A**) Representative fluorescent microscope images show cells that were fixed, permeabilized, and stained with primary antibodies against either Flag and clathrin (1∶250) using either Alexa-488 or -546 conjugated secondary antibodies; (**B**) GFP-DN-Eps-15 or (**C**) GFP-Eps-15 and Flag-CysLT_1_R transfected cells, stimulated, or not, with 80 nM LTD_4_ and stained with Flag antibody. The mounted slides were examined using a Nikon TE300 microscope (60× or 100×1.4 plan-apochromat oil immersion objective). The scale bar represents 10 µm.

We next investigated the internalization pathway of the CysLT_1_R in these cell lines. GPCRs mainly internalizes via clathrin-coated pits. Next we, therefore, investigated if the internalization of the CysLT_1_R was clathrin-dependent. Int 407 and Caco-2 cells were transiently transfected with the Flag-tagged CysLT_1_R and co-transfected with Flag and clathrin antibodies. Under basal conditions, co-localization of the CysLT_1_R with clathrin was detected at both the plasma membrane and nuclear regions (Fig. 5A). As demonstrated in both cell lines, the internalized receptor co-localized with clathrin upon LTD_4_ stimulation (Fig. 5A). In order to further confirm if the receptor was internalized via the clathrin pathway, we next used GFP-dominant negative Eps-15 (DN-Eps-15) constructs, as Eps-15 is a protein involved exclusively in the formation of clathrin-coated pits and the lack of Eps-15 activity prevents the formation of clathrin-coated vesicles [46].

Int 407 cells and Caco-2 cells were transfected with GFP-DN-Eps-15 and Flag-tagged CysLT_1_R, and stimulated, or not, with LTD_4_. The Flag-tagged CysLT_1_R was not internalized upon stimulation in cells over expressing the DN-Eps-15 construct (Fig. 5B). Conversely, in cells co-transfected with the GFP-Eps-15 construct and the Flag-CysLT_1_R receptor, internalization of the receptor upon LTD_4_ stimulation was observed (Fig. 5C). This suggests that the CysLT_1_R is internalized in a clathrin-dependent manner in both cell lines.

### CysLT_1_R Internalizes in a Rab-5- and Arrestin-3 -dependent Manner

**Figure 6:**
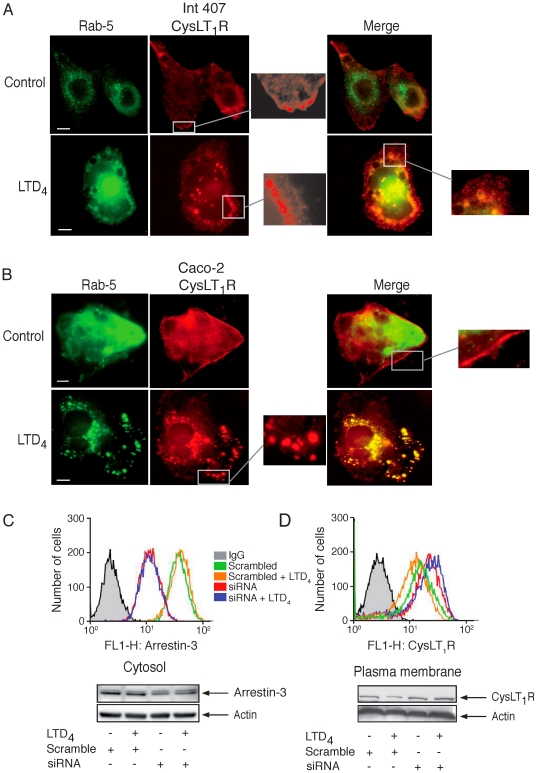
Co-localization of CysLT_1_R and Rab-5 protein in Int 407 and Caco-2 cells and arrestin-3-dependent internalization of the CysLT_1_R. Fluorescent microscope images showing cells that were fixed, permeabilized, and stained with primary antibodies against Flag (1∶2500) using Alexa-546 conjugated secondary antibodies, Flag-CysLT_1_R, and GFP-Rab-5 in Int 407 cells (**A**) and Caco-2 cells (**B**). Cells were grown on cover slips to 50-60% confluency, transfected with Flag-CysLT_1_R and GFP-Rab-5, left to rest for 48 hours, and treated with or without 80 nM LTD_4_. The mounted slides were examined using a Nikon TE300 microscope (60× or 100×1.4 plan-apochromat oil immersion objective). (**C**, **D**) Cells were transfected, or not, with siRNA against arrestin-3 or scrambled siRNA, serum-starved, and stimulated, or not, with LTD_4_ (80 nM, 5 minutes). For FACS analysis, Int 407 cells (1×10^6^ cells) were either first fixed and permabilized before intracellular staining for arrestin-3 or used directly for CysLT_1_R cell surface staining. Moreover, whole lysates or plasma membrane fractions were made and subjected to SDS-polyacrylamide gel electrophoresis and analyzed for arrestin-3 or CysLT_1_R protein expression using Western blot analysis. All membranes were re-probed for actin to ensure equal loading. The blots are representative of three separate experiments. The scale bar represents 10 µm.

The initial step after receptor internalization is the transfer of the receptors into early endosomes. Trafficking of early endosomes and clathrin-coated vesicles is often regulated by the GTPase Rab-5. Therefore, we next examined the role of Rab-5 in the CysLT_1_R internalization. Cells were co-transfected with GFP-Rab-5 and the Flag-CysLT_1_R. In unstimulated cells, the CysLT_1_R was localized at the plasma membrane (Fig. 6A, B). Upon stimulation with LTD_4_, Rab-5 positive vesicles were formed in Int 407 and Caco-2 cells and the CysLT_1_R co-localized in these vesicles (Fig. 6A, B).

GPCR internalization and desensitization is either arrestin-dependent or independent. We, therefore, proceeded to down-regulate arrestin-3 using siRNA. Treatment with siRNA resulted in an approximate 50% reduction of arrestin-3 protein expression, as demonstrated by FACS and Western blot (Fig. 6C). This reduction of arrestin-3 protein expression significantly impaired the LTD_4_-induced internalization of the CysLT_1_R (Fig. 6D). This data suggests that the CysLT_1_R is internalized in an arrestin-3-dependent manner in intestinal epithelial cells.

### CysLT_1_R Increases at the Nuclear Membrane upon LTD_4_ Stimulation

**Figure 7:**
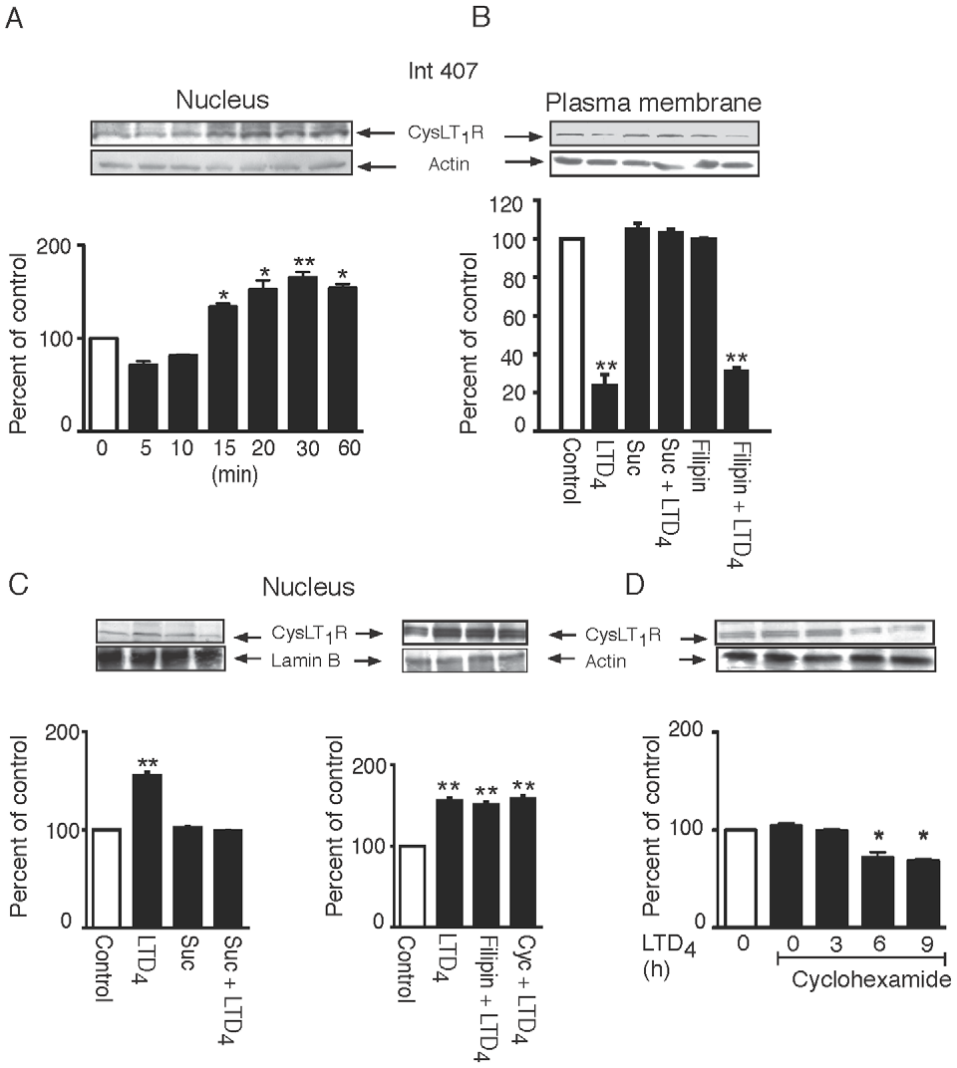
Regulation and function of CysLT_1_R at the plasma and nuclear membrane in Int 407 cells. Int 407 cells were grown to 80% confluency, serum-starved for 2 hours, stimulated, or not, with 80 nM LTD_4_, lysed, fractioned into plasma and nuclear membranes, subjected to SDS-polyacrylamide gel electrophoresis, and stained for the CysLT_1_R by Western blot. (**B–D**) Cells were pre-treated with or without sucrose, Filipin, or cycloheximide, stimulated, or not, with 80 nM LTD_4_ for 5 minutes or as indicated, lysed, fractioned into the plasma membrane (**B**) and nucleus (**A**, **C**), or whole cell lysate (**D**) and subjected to gel electrophoresis. The PDVF membranes were then stained with the CysLT_1_R antibody (1∶1000) and re-probed for actin (1∶2000) or lamin B (1∶1000) to ensure equal loading. The data are given as percent of control and represent means ± S.E.M. of at least three separate experiments. The statistical analysis was performed with a Student's *t* test. *P<0.05 and ** P<0.01.

**Figure 8:**
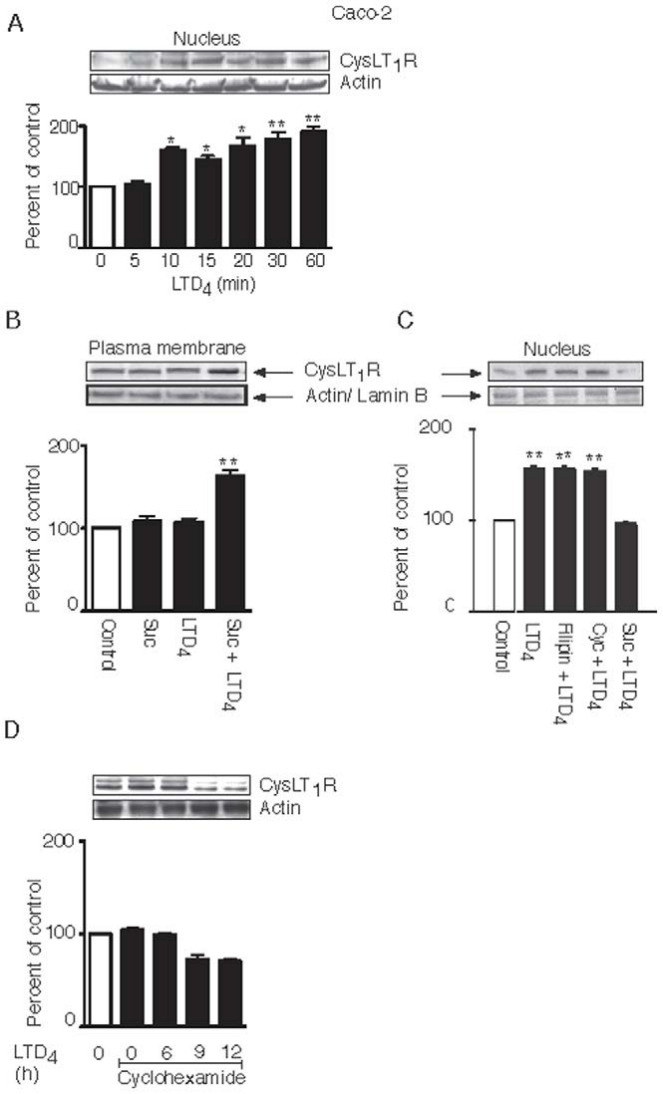
Regulation of CysLT_1_R at the plasma and nuclear membrane in Caco-2 cells. Caco-2 cells were grown to 80% confluency, serum-starved for 2 hours, stimulated, or not, with 80 nM LTD_4_, lysed, fractioned into plasma and nuclear membranes, subjected to SDS-polyacrylamide gel electrophoresis, and stained for the CysLT_1_R by Western blot. Cells were pre-treated with or without sucrose (B, **C**), Filipin (**C**), or cyclohexamide (1 hour) (**C, D**), stimulated, or not, with 80 nM LTD_4_ for 5 minutes or as indicated, lysed, fractioned into the plasma membrane (**B**) and nucleus (**A**, **C, D**), and subjected to gel electrophoresis. The PDVF membranes were then stained with the CysLT_1_R antibody (1∶1000) and re-probed for actin (1∶2000) or lamin B (1∶1000) to ensure equal loading. The data are given as percent of control and represent means ± S.E.M. of at least three separate experiments. The statistical analysis was performed with a Student's *t* test. *P<0.05 and ** P<0.01.

As previously mentioned, it has been shown that the CysLT_1_R is also localized at the nuclear membrane [12] and that the nuclear localization is increased in colorectal adenocarcinomas and facilitates survival and proliferation [12], [47]. We, therefore, investigated if the receptor at the nuclear membrane was affected by ligand stimulation. Western blot analysis of nuclear fractions of Int 407 and Caco-2 cells demonstrated that the CysLT_1_R was already significantly up-regulated after 15 minutes of LTD_4_ stimulation in Int 407 cells (Fig. 7A), after 10 minutes in Caco-2 cells (Fig. 8A), and continues to increase up to 1 hour after stimulation in both cell lines (Figs. 7A, 8A). In order to investigate if this accumulation is due to translocation of CysLT_1_R from the plasma membrane, cells were pre-treated with an inhibitor of clathrin-coated pit formation, sucrose, and as a control, we also used the caveolae inhibitor Filipin. The cells were, thereafter, fractioned into plasma and nuclear membranes, and stained with the CysLT_1_R antibody. As shown in Figure 7B, receptor internalization in Int 407 cells was blocked by the clathrin inhibitor, sucrose, but not by the caveolae inhibitor Filipin. Similarly, the increase in the nuclear fraction from the same experiment was blocked by sucrose, but not by Filipin (Fig. 7C). In Caco-2 cells, inhibiting clathrin with sucrose and stimulating the cells with LTD_4_ led to an increase of the CysLT_1_R at the plasma membrane (Fig. 8B) and, as a consequence, also inhibited the LTD_4_-induced increase at the nuclear membrane (Fig. 8C). The increase at the plasma membrane of Caco-2 cells suggests that LTD_4_ stimulation signals the recruitment of the CysLT_1_R to the plasma membrane and, when receptor internalization is blocked, it leads to a net increase of the receptor at the plasma membrane. In Int 407 cells, however, inhibiting clathrin with sucrose led to blocking receptor internalization and nuclear increase, but did not lead to an increase at the plasma membrane.

Our data demonstrates that both the internalization from the plasma membrane and the accumulation of the CysLT_1_R at the nucleus are clathrin-dependent, thus indicating that the receptor is translocating. We also explored other possibilities leading to the nuclear accumulation of the CysLT_1_R. We, therefore, investigated if the increase of the CysLT_1_R at the nuclear membrane could be due to *de novo* synthesis. However, cycloheximide (an inhibitor of protein synthesis) does not affect the increase at the nuclear membrane, suggesting that the accumulation of the CysLT_1_R is not due to new synthesis of the receptors (Figs. 7C, 8C). Furthermore, stimulation with LTD_4_ up to 1 hour did not increase CysLT_1_R expression levels in whole cell lysates (data not shown), further supporting the idea that the nuclear accumulation of CysLT_1_R is not due to new synthesis.

We have previously identified that increased expression of the CysLT_1_R in colon cancer patients correlates with a poorer prognosis [13]. Increased levels of CysLT_1_R in colon cancer cells can originate from a slower degradation of the receptor in cancer cells compared to non-transformed cells. Therefore, we next investigated the degradation of the CysLT_1_R. Cells were pre-incubated with cycloheximide and stimulated with LTD_4_ for various time points. We found a slight decrease of the CysLT_1_R after 6 hours of stimulation with LTD_4_ in Int 407 cells (Fig. 7D) and after 9 hours of stimulation in Caco-2 cells (Fig. 8D). We found it unrealistic that this small difference in receptor level could explain the increased expression level seen in colon cancer cells.

**Figure 9:**
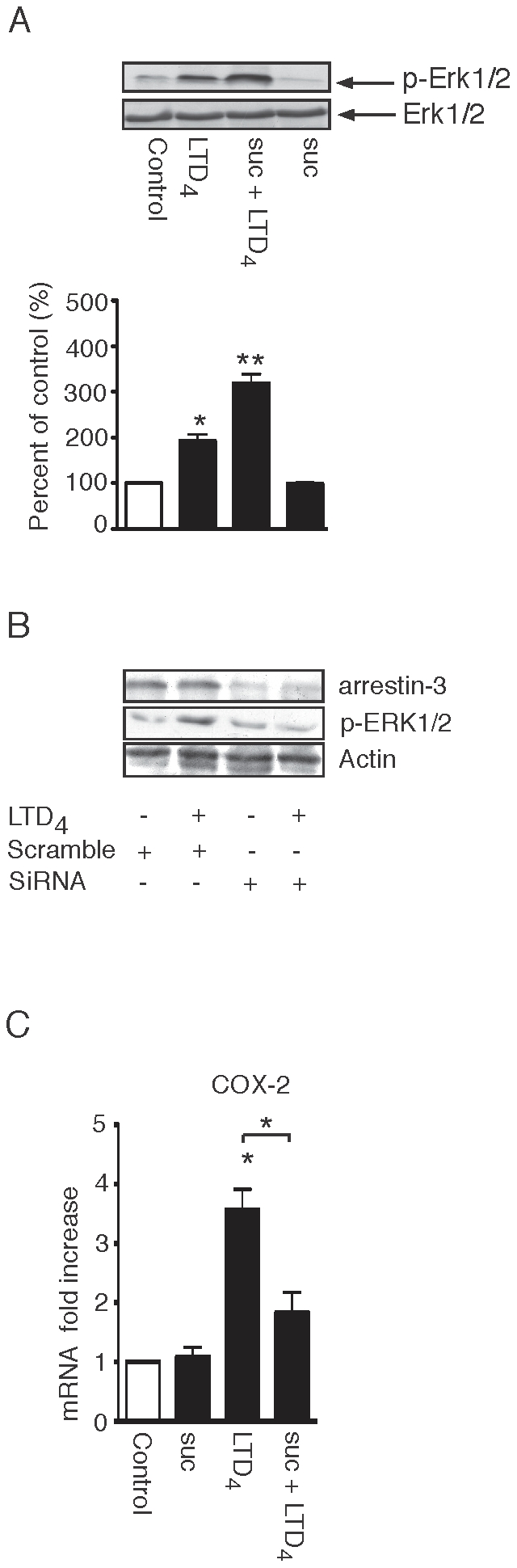
Effect of sucrose and arrestin-3 on CysLT_1_R signaling. Int 407 cells were grown to 80% confluency, serum-starved for 2 hours, pre-treated, or not, with (**A**) sucrose for 1 hour or (**B**) siRNA (arrestin-3 or scrambled) and with or without 40 nM LTD_4_ stimulation. Cell lysates were prepared as described in Methods and samples were subjected to SDS-polyacrylamide gel electrophoresis and Western blot analysis. The PDVF membranes were stained with the phospho-Erk1/2, total Erk1/2 (1∶1000), or arrestin-3 and actin (1∶2000) antibodies. All membranes were re-probed for actin to ensure equal loading. (**C**) shows Q-PCR of COX-2 mRNA from Int 407 cells pre-treated, or not, with sucrose and stimulated, or not, with LTD_4_ (80 nM, 1 hour). The RT-PCR was performed as described in Methods, using primers for COX-2. The data are given as fold increase compared to control and represent means ± S.E.M. The statistical analysis was performed with a Student's *t* test. *P<0.05 and ** P<0.01.

We have previously demonstrated that LTD_4_ via the CysLT_1_R induces Erk1/2 phosphorylation [9]. We have now shown that blocking the internalization of the CysLT_1_R does not reduce this phosphorylation but, instead, a slight increase of the signal is detected (Fig. 9A). Arrestins are also involved in Erk1/2 signaling downstream of GPCRs [48]. We, therefore, investigated the potential functional effect of arrestin-3 knockdown on CysLT_1_R signaling. Here we demonstrated that down-regulating arrestin-3 decreases LTD_4_-induced Erk1/2 phosphorylation (Fig. 9B). We next investigated the potential functional effect of clathrin inhibition by sucrose on CysLT_1_R signaling. We stimulated cells with LTD_4_ with, or without, sucrose and investigated the effect on one of the target genes for CysLT_1_R, COX-2. We found that sucrose decreases the LTD_4_-induced mRNA level of COX-2 (Fig. 9C); this data suggests that in contrast to Erk1/2 phosphorylation, the internalization of the receptor is important for activation of the *COX*-2 gene.

## Discussion

GPCRs have been extremely successful drug targets for a multitude of diseases [49], [50] as with the CysLT_1_R antagonist, Montelukast, which is currently used as a treatment for asthma [51]. The assembly of GPCRs as homo- and hetero-oligomers and their phosphorylation and association with a vast array of trafficking and signal-modulating proteins are emerging as major mechanisms underlying the functioning of GPCRs. It has become increasingly evident that GPCR signaling, expression, localization, and trafficking often play a role in disease development and progression [52]. One example is retinitis pigmentosa, which results from improper intracellular trafficking and localization of the rhodopsin receptors [19], [20]. Furthermore, previous studies by our group show that increased nuclear expression of the CysLT_1_R correlates with a poorer prognosis for colon cancer patients [12], [13]. Here, we wanted to investigate the trafficking of the CysLT_1_R, which is a major regulatory mechanism of GPCR signaling. Previous studies have shown that LTD_4_ binds the CysLT_1_R with a higher affinity than LTC_4_ [3]. In this study, we conclude that LTC_4_ and LTD_4_ affect the trafficking of CysLT_1_R differently, suggesting ligand-specific signaling. We also demonstrate that LTD_4_ mainly internalizes the CysLT_1_R, supporting our previous findings that LTD_4_-induced cell survival, cell proliferation, and cell migration are mediated through the CysLT_1_R. This is further supported by the fact that LTD_4_ stimulation decreases the dimerization observed between the CysLT_1_R and CysLT_2_R, as demonstrated by the *in situ* proximity ligation assay and immunoprecipitation data. It is interesting to note that after 60 minutes of stimulation with LTD_4_, the amount of heterodimers observed is concentrated in the nuclear region, supporting the results of nuclear accumulation of the CysLT_1_R. The LTC_4_, on the other hand, does not lead to a nuclear accumulation of either receptor, but induces internalization of both CysLT_1_ and CysLT_2_ receptors, which might be due to the preserving of the receptor dimers. Another interesting observation was the effect of the ligand-induced tyrosine phosphorylation of CysLT_1_R. LTD_4_ induced tyrosine phosphorylation of the CysLT_1_R, but not of the CysLT_2_R, which clearly shows the specificity of the ligand-induced signaling. This correlates well with the fact that CysLT_1_R is the high affinity receptor and affects cell proliferation, survival, and cell migration, whereas CysLT_2_R does not [8], [9]. Moreover, these results also support our previous findings that inhibition of the CysLT_1_R leads to growth inhibition and cell death [47]. The CysLT_2_R has been shown to be a negative regulator of the mitogenic effect of the CysLT_1_R upon LTD_4_ stimulation in mast cells [53].

We found that in Int 407 cells, the internalization of the CysLT_1_R could be detected after 5 minutes of stimulation, which could be blocked by a specific CysLT_1_R antagonist. However, in Caco-2 cells, the endogenous internalization was more difficult to detect. This may be due to a high turnover of the receptor at the plasma membrane upon stimulation. This hypothesis is supported by the fact that internalization blocking experiments lead to an accumulation of the receptor at the plasma membrane, which cannot be seen in Int 407 cells. However, using FACS analysis with the Caco-2 cells, we could detect a small but significant internalization of the endogenous receptors after LTD_4_ stimulation, supporting that there is an internalization of the receptor.

We next investigated how the CysLT_1_R is internalized. We found that CysLT_1_ is internalized in a clathrin/Rab-5-dependent pathway; we also suggest that this internalization is arrestin-3-dependent. Previous publication studying CysLT_1_R internalization in other cells has demonstrated that this process is arrestin-3 independent [16]. In that study, dominant negative (DN)-arrestin-3 constructs were used in HEK-293 cells. The results demonstrated that when arrestin-3 was over expressed, receptor internalization was increased. However, over expression with a dominant negative construct lead to a non-significant decrease of receptor internalization. These results were further supported by experiments in MEF cells from arrestin-3 deficient mice [16]. To conclude, whether CysLT_1_R required arrestin to internalize or not, we used siRNA against endogenous arrestin-3 and demonstrated that the loss of the receptor from the plasma membrane is inhibited. Our results show that CysLT_1_R requires arrestin-3 for internalization in epithelial cells. Furthermore, GPCRs can activate Erk1/2 in an arrestin-dependent manner [48]. As shown in our results, down-regulation of arrestin-3 disrupts LTD_4_-induced Erk1/2 phosphorylation. In summary, these data demonstrate that the CysLT_1_R is internalized from the plasma membrane in a clathrin-, Rab-5-, and arrestin-3-dependent manner and that inhibition of arrestin-3 also affects the signaling downstream of the CysLT_1_R.

As previously shown, the CysLT_1_R is also localized at the nuclear membrane. We demonstrated here that the increase of the receptor at the nuclear membrane coincides with the loss from the plasma membrane. Both the accumulation and the cell surface loss of the CysLT_1_R are clathrin-dependent. The accumulation of the CysLT_1_R at the nucleus is not due to new synthesis of the receptor, although there is a possibility of the existence of an internal pool of the receptor that could be responsible for the accumulation. In fact, an internal pool could be one possibility of receptor recruitment to the plasma membrane and a high turnover of CysLT_1_R upon LTD_4_ stimulation in Caco-2 cells. In order to investigate the role of the nuclear accumulation of the CysLT_1_R, we investigated the signaling of the receptor. By inhibiting the formation of clathrin-coated pits and, thereby, inhibiting receptor accumulation at the nuclear membrane, LTD_4_-induced COX-2 mRNA up-regulation was decreased. This suggests that the CysLT_1_R accumulation at the nucleus, or its internalization, is required for certain signaling pathways.

Taken together, our results show how the receptor is trafficking from the plasma membrane to the nucleus and demonstrates different regulation of CysLT_1_R signaling.

## Supporting Information

Figure S1 Co-Immunoprecipitation of the CysLTRs in colon cancer cells. Briefly HCT-116 cells were grown to 80% confluency and lysed. Lysates containing 1 mg/ml protein were incubated with rabbit anti-CysLT_2_R antibody, after which 20 µg of protein A plus agarose was added. The beads then were washed three times mixed with sample buffer, boiled and centrifuged. The proteins were then separated on SDS-polyacrylamide gels. The separated proteins were electrophoretically transferred to a polyvinylidene difluoride (PDVF) membrane incubated with a primary antibody against CysLT1R overnight. Thereafter the membrane was exposed to hyperfilm-ECL to visualize immunoreactive proteins. The membrane was then re-probed with a CysLT_2_R antibody. The blots shown are representative and the data are given as percent of control and represents means ± S.E.M. of three separate experiments and the statistical analysis were performed with Student's t test. *P<0.05.
(0.36 MB TIF)

Figure S2 The internalization of CysLT_1_R and CysLT_2_R after LTC4 stimulation. Int 407 cells were grown to 80% confluency and then treated with or without 40 nM LTC4 for indicated periods of time. Plasma membrane fractions were prepared as described in Materials and Methods and samples were subjected to SDS-polyacrylamide gel electrophoresis and Western blot analysis. The PDVF membranes were stained with CysLT_1_R, CysLT_2_R (both 1∶1000) or actin (1∶2000) antibodies. The blots shown are representative and the data are given as percent of control and represents means ± S.E.M. of three separate experiments and the statistical analysis were performed with Student's t test. *P<0.05 and ** P<0.01.
(0.08 MB TIF)
