# Supplementary figures and images for: Ligand-Induced Tyrosine Phosphorylation of Cysteinyl Leukotriene Receptor 1 Triggers Internalization and Signaling in Intestinal Epithelial Cells
Source: PLoS One. 2010 Dec 28;5(12):e14439. doi: 10.1371/journal.pone.0014439 (PMC3010979; doi:10.1371/journal.pone.0014439)

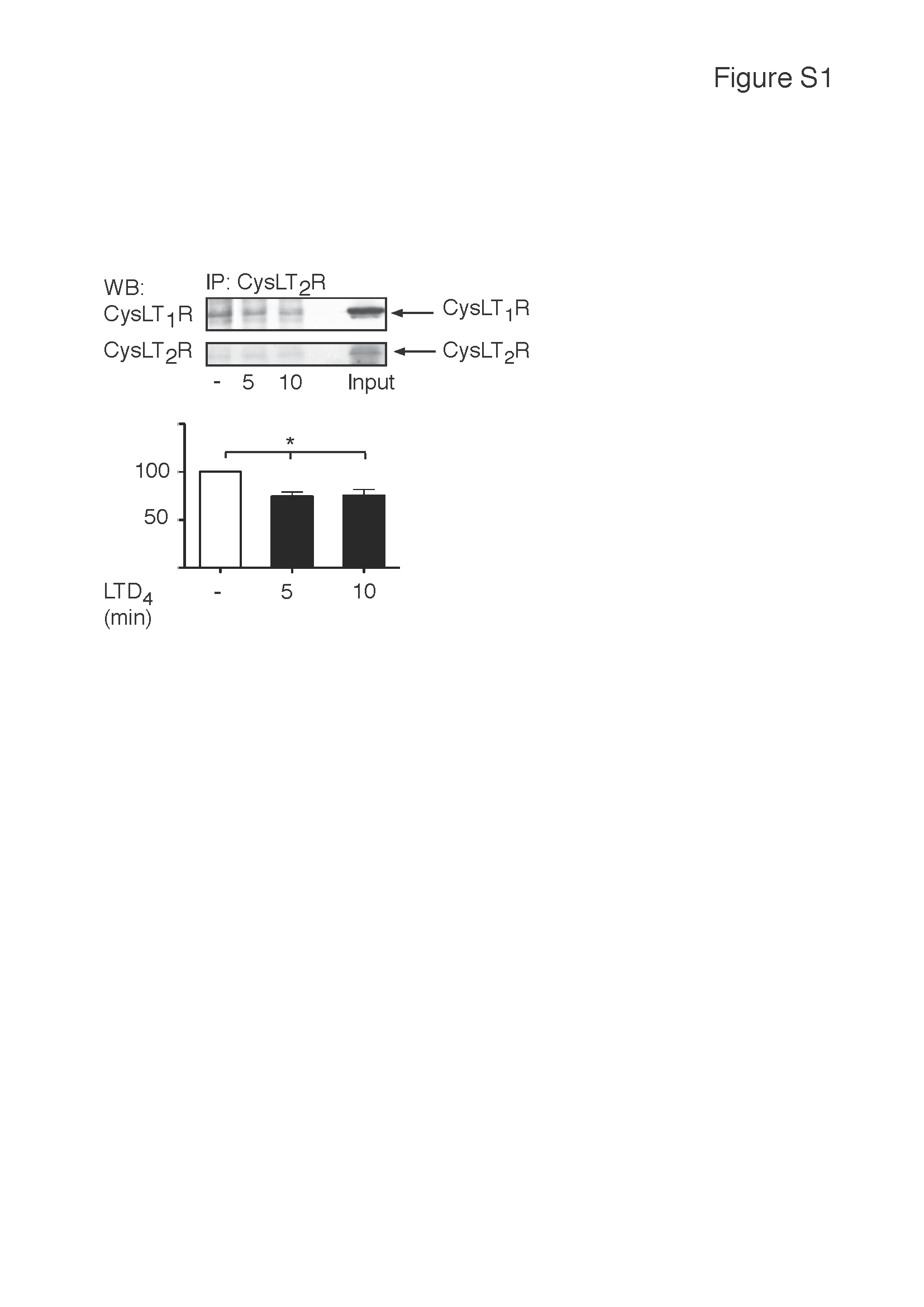

Supplement: Figure S1 — Co-Immunoprecipitation of the CysLTRs in colon cancer cells. Briefly HCT-116 cells were grown to 80% confluency and lysed. Lysates containing 1 mg/ml protein were incubated with rabbit anti-CysLT2R antibody, after which 20 µg of protein A plus agarose was added. The beads then were washed three times mixed with sample buffer, boiled and centrifuged. The proteins were then separated on SDS-polyacrylamide gels. The separated proteins were electrophoretically transferred to a polyvinylidene difluoride (PDVF) membrane incubated with a primary antibody against CysLT1R overnight. Thereafter the membrane was exposed to hyperfilm-ECL to visualize immunoreactive proteins. The membrane was then re-probed with a CysLT2R antibody. The blots shown are representative and the data are given as percent of control and represents means ± S.E.M. of three separate experiments and the statistical analysis were performed with Student's t test. *P<0.05. (0.36 MB TIF) [file pone.0014439.s001.tif]

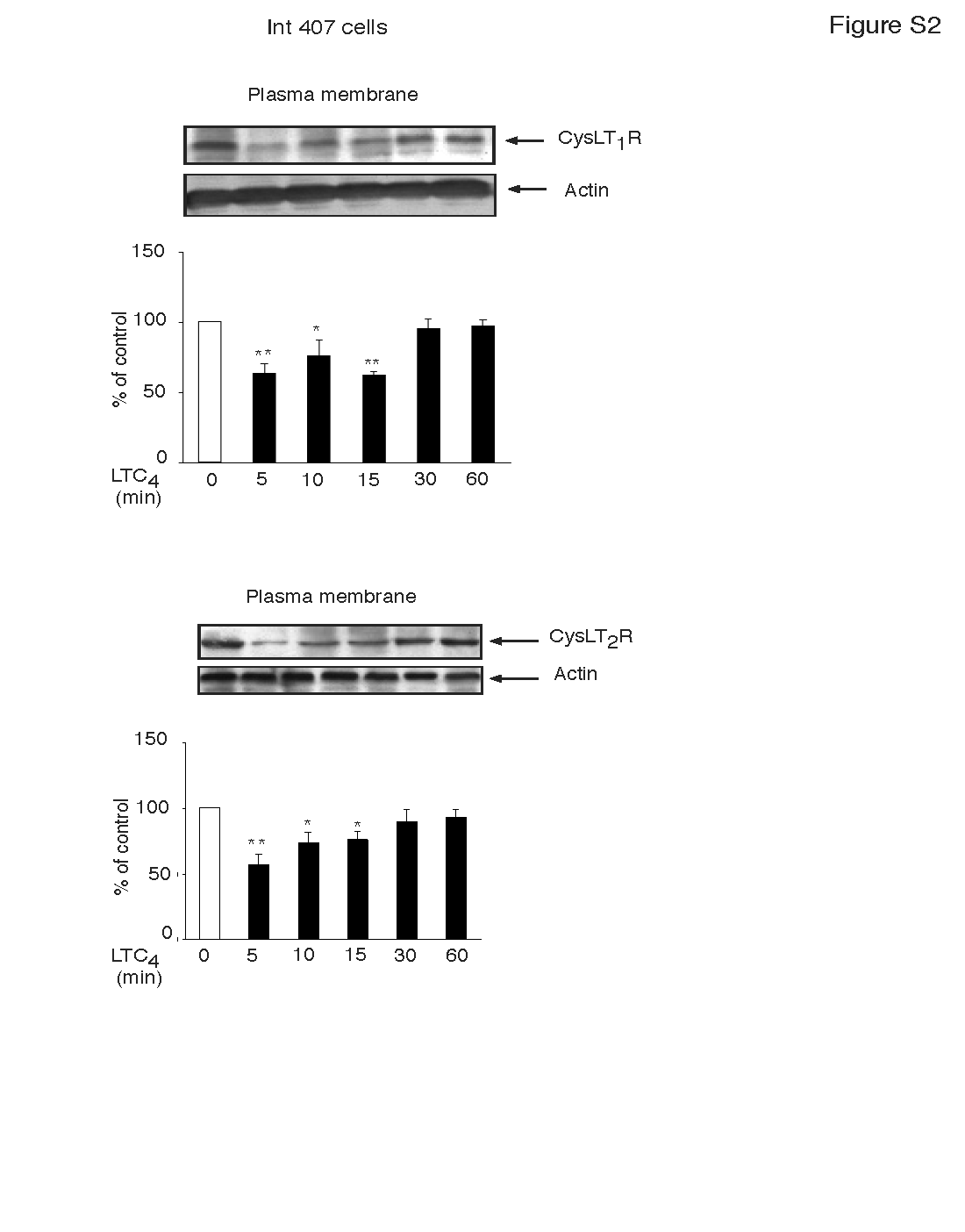

Supplement: Figure S2 — The internalization of CysLT1R and CysLT2R after LTC4 stimulation. Int 407 cells were grown to 80% confluency and then treated with or without 40 nM LTC4 for indicated periods of time. Plasma membrane fractions were prepared as described in Materials and Methods and samples were subjected to SDS-polyacrylamide gel electrophoresis and Western blot analysis. The PDVF membranes were stained with CysLT1R, CysLT2R (both 1∶1000) or actin (1∶2000) antibodies. The blots shown are representative and the data are given as percent of control and represents means ± S.E.M. of three separate experiments and the statistical analysis were performed with Student's t test. *P<0.05 and ** P<0.01. (0.08 MB TIF) [file pone.0014439.s002.tif]
